# Targeting Neuropeptide Y/DPP4 Signalling Suppresses Ewing Sarcoma Survival and Improves Monocyte Viability

**DOI:** 10.3390/ijms27062731

**Published:** 2026-03-17

**Authors:** Robin M. H. Rumney, Dariusz C. Górecki

**Affiliations:** School of Medicine, Pharmacy and Biomedical Sciences, University of Portsmouth, Portsmouth PO1 2DT, UK

**Keywords:** BMS-193885, dipeptidyl peptidase-4, Ewing sarcoma, hypoxia, linagliptin, neuropeptide Y

## Abstract

Survival rates for metastatic Ewing sarcoma (EwS) have remained persistently low over recent decades, highlighting the need for more effective chemotherapeutic options. Potential targets may be found within the Neuropeptide Y (NPY) signalling pathway that has been implicated in EwS cell survival. However, confounding factors include hypoxia that modulates NPY signalling, dipeptidyl peptidase-4 (DPP4/CD26) that cleaves NPY and interactions via NPY signalling from infiltrating immune cells. We investigated these interactions in A673 and SK-ES-1 EwS cell lines and THP-1 monocytes to identify therapeutic targets suitable for drug repurposing. Both EwS cell lines secreted NPY into conditioned media and extracellular vesicles. Recombinant NPY enhanced viability of both A673 and SK-ES-1 cells; however, the NPY1R antagonist BMS-193885 reduced viability in A673 cells only. Recombinant DPP4 widely promoted EwS viability and, under hypoxic conditions, it increased cell metabolism. The DPP4 inhibitor linagliptin, which is used clinically, consistently suppressed EwS viability with elevated sensitivity under hypoxia, where there was increased cell death of SK-ES-1 cells. Conversely, in THP-1 monocytes, NPY suppressed metabolism, BMS-193885 increased live-cell staining and DPP4 induced cell death. These findings suggest that NPY and DPP4 enhance EwS survival through autocrine/paracrine signalling while reducing monocyte viability. Thus, targeting the NPY/DPP4 signalling axis may provide therapeutic benefit by directly suppressing EwS growth and enhancing efficacy of immunotherapy.

## 1. Introduction

Ewing sarcoma (EwS) is the second most common primary bone cancer after osteosarcoma, making up 14% of all primary bone tumours and ~1% of all childhood cancers [1]. Five-year survival rates for Ewing sarcoma (EwS) are around 70–80% in non-metastatic cases, dropping to 20–25% when distant metastases are present [2]. Approximately 20–30% of patients present with metastatic disease [3], and metastasis of EwS tumours remains the most important factor in determining patient survival [4].

EwS was previously thought to have a neural histogenesis related to primitive neuroectodermal tumours [5], but it is now well established that EwS has an origin in mesenchymal stem cells (MSCs) [6]. The trigger for EwS formation comes from a chromosomal translocation event that, in 85% of cases, occurs between Ewing sarcoma breakpoint region 1 (EWSR1) and Friend leukaemia virus integration 1 (FLI1), resulting in the EWS-FLI1 fusion oncogene, which drives many of the pathways associated with the disease [7], including those associated with proliferation and survival [7,8].

Current treatments for EwS rely upon combined radiotherapy, surgery and chemotherapy [9]. Dose-intensive chemotherapy with vincristine, doxorubicin, cyclophosphamide, ifosfamide, and etoposide has proven effective against early-stage, non-metastatic EwS [10].

However, it has proven far more challenging to find chemotherapeutic treatments for metastatic EwS, with several potential treatments reaching Phase II and Phase III clinical trials [11,12], only to prove ineffective against the metastatic illness. The current situation underscores a requirement for new targets that might enable more effective management.

One signalling pathway that has received recent attention involves extracellular neuropeptide Y (NPY). Serum levels of NPY have been found elevated in patients with EwS [13], while hypoxia and increased expression of NPY in EwS cells have been linked to metastasis [14,15]. NPY acts upon cell surface NPY receptors with a modulating role for Dipeptidyl peptidase-4 (DPP4, also known as CD26), which cleaves NPY and controls signalling as specific receptors have different binding affinities for the full-length and cleaved NPY molecule [13].

Exogenous NPY also drives changes in macrophage activity by promoting migration [16] and infiltration [17], while inducing M1 inactivation [18] and a switch towards M2 polarisation [19]. NPY release by macrophages has been reported [20], and NPY is involved in crosstalk between different cell types, for example, between adipocytes and macrophages [19]. This raises the intriguing possibility that NPY signalling may be involved in crosstalk between EwS cells and macrophages. Such crosstalk between different cell populations may offer a potential explanation for why EwS has an immunosuppressive tumour microenvironment, allowing for tumour immune escape that has stifled attempts at immunotherapy [21,22].

Drug repurposing minimises the need for expensive clinical trials and offers a practical strategy to bridge the gap from “bench to bedside” in a more cost-effective and timely manner, which would be particularly desirable for rare diseases such as EwS. Indeed, there are several repurposed drugs that have shown encouraging results in slowing cancer progression. Thalidomide, originally developed as a treatment for morning sickness, is now used to treat multiple myeloma, where it has been shown to improve progression-free survival [23]. The beta-blocker Propranolol is effective against a broad range of cancer cells *in vitro* and *in vivo* and has been clinically associated with a decrease in all cancer mortality [24]. Mebendazole was developed as an anti-parasitic treatment that has since been shown to be effective against breast cancer cells *in vitro* and *in vivo* [25], and it has reached Phase 2a clinical trials for the treatment of gastrointestinal cancer [26]. The diabetes medication Metformin may also have some beneficial effects in treating cancer [27,28]. These examples demonstrate that drug repurposing is a potentially valuable strategy for cancer treatment, providing a sound rationale for attempting such an approach with EwS.

In this study, we hypothesised that NPY–DPP4 signalling supports Ewing sarcoma cell survival under normoxic and hypoxic conditions while suppressing monocyte viability, and that pharmacological inhibition of this pathway would reduce tumour viability and improve immune-supportive responses. To investigate this, we conducted an in-depth analysis of the NPY–DPP4 axis in two widely used EwS cell lines to determine whether targeting this signalling pathway could reduce EwS cell viability and potentially lead to improved therapeutic strategies. Specifically, we investigate NPY1R receptor antagonists developed for the treatment of obesity [29] and gliptins, which are medicines developed to treat diabetes by inhibition of DPP4 [30]. We also investigate the effects of NPY and DPP4 upon THP-1 monocytes to identify potential interactions between EwS cells, which could explain the immunosuppressive mechanisms reflected in EwS tumours [22], and discuss what the implications are of this for future immunotherapeutic options.

## 2. Results

### 2.1. Neuropeptide Y Release from EwS Cells

**Figure 1:**
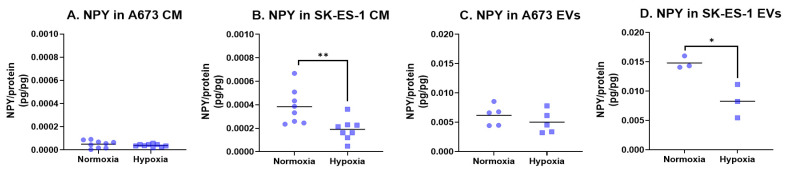
NPY secretion by EwS cells. NPY was quantified from A673 and SK-ES-1 cells after 48 h in serum-free media in normoxia and hypoxia. NPY protein expression in cell-conditioned media (CM) was quantified from (**A**) A673 and (**B**) SK-ES-1 cells (** = *p* < 0.01, *t*-test). NPY protein expression was quantified from extracellular vesicles (EVs) from (**C**) A673 and (**D**) SK-ES-1 cells (* = *p* < 0.05, *t*-test).

*NPY* transcript levels were quantified by qPCR and normalised to β-actin (*ACTB*) using the ΔΔCt method. *NPY* transcript expression did not differ between normoxic and hypoxic conditions in either A673 or SK-ES-1 cells (Supplementary Figure S1A,B). Consistent with this, A673 cells released low levels of NPY into conditioned media (40.7 ± 24.6 pg/mL) under normoxia, with no significant change in NPY/protein vlaues in hypoxia (Figure 1A; *n* = 9 from three independent experiments). In contrast, SK-ES-1 cells secreted markedly higher levels of NPY (337.9 ± 132.4 pg/mL under normoxia), which decreased to 257.0 ± 113.6 pg/mL in hypoxia. NPY/protein values were reduced by approximately 50% under hypoxic conditions (Figure 1B, *p* < 0.01; *n* = 8 from three independent experiments). These data indicate that *NPY* transcript levels do not predict NPY protein secretion and that SK-ES-1 cells exhibit hypoxia-sensitive NPY release.

To assess whether hypoxia alters the mode of NPY release, extracellular vesicle (EV)-associated NPY was quantified by ELISA and normalised to EV protein content. In A673 cells, EV-associated NPY/protein (0.006 pg/pg) was unchanged by hypoxia (Figure 1C; *n* = 5). In SK-ES-1 cells, EV-associated NPY/protein decreased by 43% from 0.0148 pg/pg under normoxia to 0.0083 pg/pg in hypoxia (Figure 1D; *p* < 0.05; *n* = 3), indicating reduced NPY output rather than altered release mechanisms.

### 2.2. Effects of rhNPY Treatment

**Figure 2:**
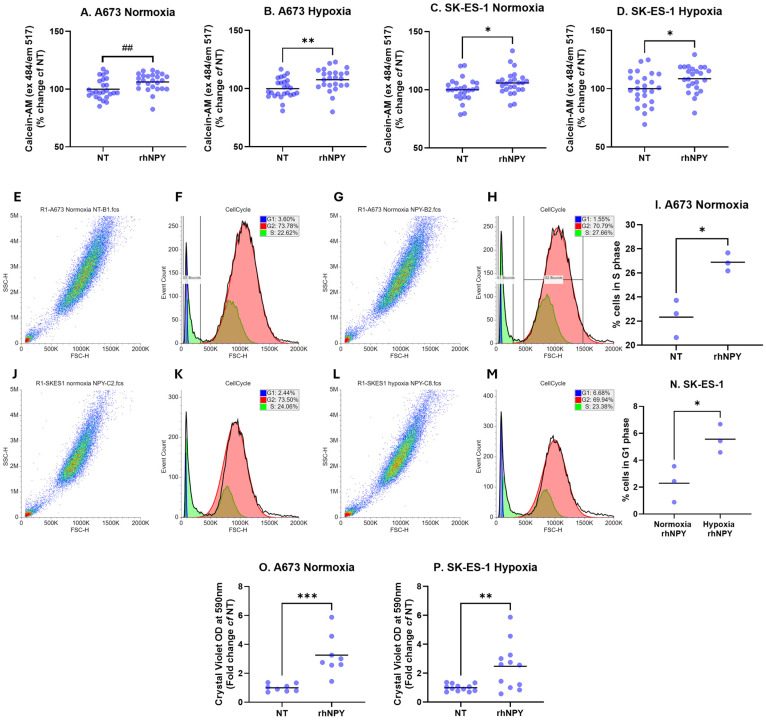
Effects of rhNPY upon EwS cells. A673 and SK-ES-1 cells were treated with 1 ng/mL rhNPY for 48 h under normoxic or hypoxic conditions in serum-free media. Live-cell staining was assessed using Calcein-AM in (**A**) A673 cells in normoxia and (**B**) hypoxia; and in SK-ES-1 cells in (**C**) normoxia and (**D**) hypoxia (*n* = 25 per condition from three independent experiments). rhNPY increased the proportion of (**E**–**I**) A673 cells in S phase under normoxia and (**J**–**N**) SK-ES-1 cells in G1 phase under hypoxia (*n* = 3 independent experiments). rhNPY also enhanced Transwell migration of (**O**) A673 cells under normoxia (*n* = 8 per condition from two independent experiments) and (**P**) SK-ES-1 cells under hypoxia (*n* = 12 per condition from three independent experiments) (* = *p* < 0.05, ** = *p* < 0.01, *** = *p* < 0.001, *t*-test; ## *p* < 0.01, Mann–Whitney test). NT—nontreated controls.

Next, we investigated the impact of NPY axis activation. Treatment with 1 ng/mL rhNPY for 48 h in serum-free media increased Calcein-AM live cell staining of A673 cells in normoxia by 6.2% (Figure 2A, *p* < 0.01, Mann–Whitney test) and in hypoxia by 7.6% (Figure 2B, *p* < 0.01, *t*-test) compared to nontreated controls. Similarly, live cell staining in SK-ES-1 in normoxia was increased by 5.8% (Figure 2C, *p* < 0.05, *t*-test), and in hypoxia by 8.7% (Figure 2D, *p* < 0.05, *t*-test, *n* = 25 per condition from three independent experiments).

The remaining effects of rhNPY were cell line and hypoxia-specific. Under normoxic conditions, rhNPY increased the proportion of A673 cells in S phase (Figure 2E–H, representative flow cytometry images and cell cycle quantification from Floreada.io; quantification in Figure 2I, *p* < 0.05, *t*-test; *n* = 3 independent experiments). SK-ES-1 cells were more responsive in hypoxia, exhibiting a higher proportion of cells in G1 phase (Figure 2J–M, representative flow cytometry images and cell cycle quantification from Floreada.io; quantification in Figure 2N, *p* < 0.05, *t*-test; *n* = 3 independent experiments).

rhNPY also enhanced transwell migration of EwS cell lines in a hypoxia-dependent manner, increasing migration of A673 cells under normoxia (Figure 2O and Supplementary Figure S2A; *p* < 0.01; *n* = 8 from two independent experiments) but not hypoxia (Supplementary Figure S2B). Meanwhile, there were no significant effects of rhNPY on SK-ES-1 migration in normoxia (Supplementary Figure S2C), but there was a significant increase under hypoxia (Figure 2P and Supplementary Figure S2D; *p* < 0.05; *n* = 12 from three independent experiments). Thus, exogenous rhNPY increases EwS cell viability as well as modulating the cell cycle and migration, suggesting autocrine effects for endogenously secreted NPY.

### 2.3. Cell-Specific Impact of NPY1R Activation

*NPY1R* and *NPY5R* transcript expression were measured by qPCR and normalised to *ACTB* with the ΔΔCt method. *NPY1R* was unaltered by hypoxia in both EwS cell lines (Supplementary Figure S3A,B); *NPY5R* was unchanged in A673 cells (Supplementary Figure S3C) but was decreased by hypoxia in SK-ES-1 cells (Supplementary Figure S3D). Immunofluorescent labelling revealed punctate staining for NPY1R protein in A673 cells in both normoxia (Supplementary Figure S3E) and hypoxia (Supplementary Figure S3F); however, no labelling for NPY1R was detected on SK-ES-1 cells.

Concordant with the expression of NPY1R in A673 cells, treatment with the NPY1R antagonist BIBP3226 in normoxia decreased crystal violet (CV) staining of A673 cells by 10% while BMS-193885 caused a 16% decrease (Supplementary Figure S3G, *p* < 0.01 and *p* < 0.0001 respectively, Kruskal-Wallis with Dunn’s multiple comparison tests, *n* = 25 from three independent experiments). BMS-193885 also decreased metabolism of A673 cells by 12% (Supplementary Figure S3H, *p* < 0.05, ANOVA with Dunnett’s multiple comparison tests, *n* = 10 from two independent experiments), with no similar effects on SK-ES-1 cells.

Thus, the effects of pharmacological NPY1R inhibition are limited, suggesting that targeting NPY1R may confer clinical benefit only in NPY1R-expressing EwS subtypes under normoxic conditions.

### 2.4. DPP4 Expression and Activity in EwS Cells

**Figure 3:**
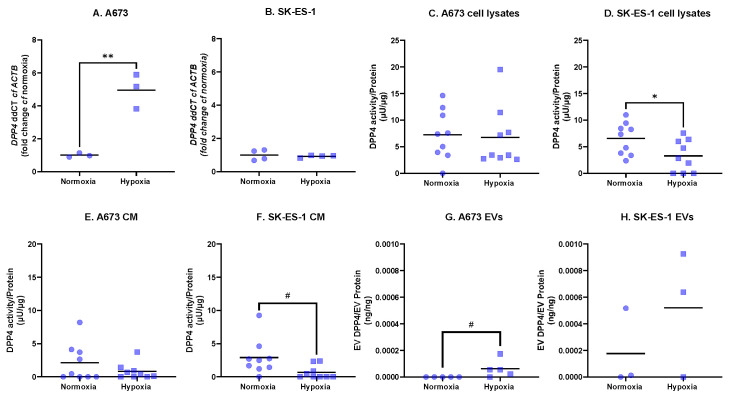
DPP4 expression. Transcript expression of *DPP4*, protein expression of DPP4 and enzymatic activity of DPP4 were quantified from A673 and SK-ES-1 after 48 h in serum-free media in normoxia and hypoxia. *DPP4* expression in (**A**) A673 cells and (**B**) SK-ES-1 cells in normoxia and hypoxia (** = *p* < 0.01, *t*-test, *n* = 3–4 independent experimental replicates). DPP4 enzymatic activity in lysates from (**C**) A673 cells and (**D**) SK-ES-1 cells in normoxia and hypoxia (* = *p* < 0.05, *t*-test). DPP4 enzyme activity in cell-conditioned media (CM) from (**E**) A673 and (**F**) SK-ES-1 cells in normoxia and hypoxia (# = *p* < 0.05, Mann–Whitney test). DPP4 protein in extracellular vesicles (EVs) from (**G**) A673 and (**H**) SK-ES-1 cells in normoxia and hypoxia (# = *p* < 0.05, Mann–Whitney test).

*DPP4* transcript expression was also measured using the ΔΔCt method. Hypoxia increased *DPP4* transcript expression in A673 cells 3.96-fold (Figure 3A, *p* < 0.01, *t*-test, *n* = 3 independent replicates), with no effect in SK-ES-1 cells (Figure 3B). DPP4 activity in cell lysates and cell-conditioned media (CM) was quantified via enzymatic assay and normalised to protein from corresponding lysates. DPP4 activity in A673 cell lysates under normoxia was 7.2 + 4.7 μU/μg, with no change in hypoxia (Figure 3C, *n* = 9 from three independent experiments). DPP4 activity in SK-ES-1 cell lysates was 6.5 + 3.0 μU/μg in normoxia and decreased by 50% to 3.3 + 3.0 μU/μg in hypoxia (Figure 3D, *p* < 0.05, *t*-test, *n* = 9 from three independent experiments). DPP4 activity in A673 CM was 2.1 + 2.8 μU/μg in normoxia with no significant change in hypoxia (Figure 3E), whereas in SK-ES-1 CM, DPP4 activity was 2.9 + 2.7 μU/μg in normoxia and was 77% lower in hypoxia at 0.7 + 1.0 μU/μg (Figure 3F, *p* < 0.05, Mann–Whitney test, *n* = 9 from three independent experiments). No DPP4 activity was detectable in EV samples. DPP4 protein measured by ELISA and normalised to corresponding protein samples was not found in EVs from A673 cells cultured under normoxia but increased to 6.1 + 6.7 × 10^−5^ ng/ng in hypoxia (Figure 3G, *p* < 0.05, Mann Whitney test, *n* = 5 independent experiments). In SK-ES-1 EVs there was 1.7 + 2.9 × 10^−4^ ng/ng in normoxia, but no significant difference was found in hypoxia (Figure 3H, *n* = 3 independent experiments). Thus, hypoxia-induced increases in DPP4 transcript expression in A673 cells correlate with increased DPP4 protein levels in A673 EVs; however, DPP4 enzymatic activity is regulated independently of these in a cell-specific manner.

### 2.5. Effect of Exogenous rhDPP4

**Figure 4:**
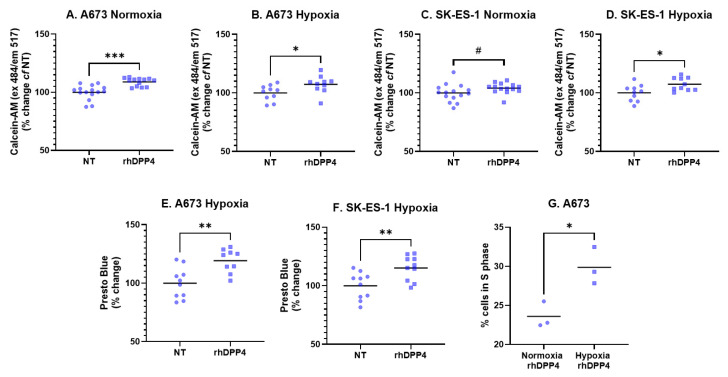
Effects of rhDPP4. A673 and SK-ES-1 cells were treated with 20U/mL rhDPP4 in normoxia and hypoxia for 48 h. Live cell staining with Calcein-AM was increased by rhDPP4 in (**A**) A673 cells in normoxia; (**B**) A673 cells in hypoxia; (**C**) SK-ES-1 cells in normoxia; (**D**) SK-ES-1 cells in hypoxia. rhDPP4 increased NADH metabolism in (**E**) A673 cells and (**F**) SK-ES-1 cells in hypoxia. (**G**) Treatment with rhDPP4 in hypoxia also increased the percentage of A673 cells in S phase (* = *p* < 0.05, ** = *p* < 0.01, *** = *p* < 0.001 *t*-test; # = *p* < 0.05 Mann–Whitney test). NT—nontreated controls.

Live-cell staining of EwS cells was consistently increased by rhDPP4 treatment. In A673 cells, staining increased by 8.9% under normoxia (Figure 4A; *p* < 0.001, *t*-test; *n* = 13–15 per condition from three independent experiments) and by 7.3% under hypoxia (Figure 4B; *p* < 0.05, *t*-test; *n* = 10 per condition from two independent experiments). In SK-ES-1 cells, rhDPP4 increased live-cell staining by 4.0% under normoxia (Figure 4C; *p* < 0.05, Mann–Whitney test; *n* = 14–15 per condition from three independent experiments) and by 7.4% under hypoxia (Figure 4D; *p* < 0.05, *t*-test; *n* = 10 per condition from two independent experiments).

rhDPP4 had no effect on cellular metabolism under normoxic conditions. However, under hypoxia, rhDPP4 increased metabolism by 19.2% in A673 cells (Figure 4E; *p* < 0.01, *t*-test; *n* = 10 per condition from two independent experiments) and by 15.2% in SK-ES-1 cells (Figure 4F; *p* < 0.01, *t*-test; *n* = 10 per condition from two independent experiments). rhDPP4 did not affect transwell migration (Supplementary Figure S2A–D). A cell line-specific effect was observed in which hypoxia sensitised A673 cells to rhDPP4, resulting in an increased proportion of cells in S phase (Figure 4G; *p* < 0.05, *t*-test; *n* = 3 independent experiments), indicating increased proliferation.

Collectively, these data indicate that DPP4 supports EwS cell survival under both normoxic and hypoxic conditions, with hypoxia-specific effects on metabolism but no increase in invasive potential.

### 2.6. Effect of DPP4 Inhibition on EwS Cell Viability

**Figure 5:**
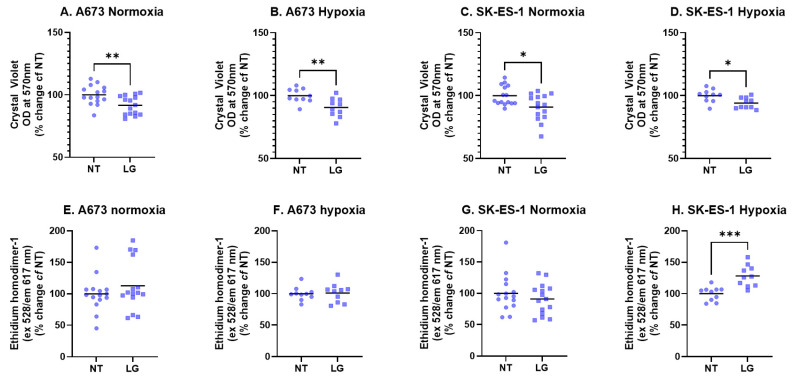
Effect of DPP4 inhibition. A673 and SK-ES-1 cells were treated with 1ng/mL linagliptin (LG) in normoxia and hypoxia for 48 h. Crystal violet staining was quantified from A673 cells in (**A**) normoxia and (**B**) hypoxia, and SK-ES-1 cells in (**C**) normoxia and (**D**) hypoxia (* = *p* < 0.05, ** = *p* < 001, *t*-test). Cell death was quantified with the cell death marker Ethidium homodimer-1 from (**E**) A673 cells in normoxia; (**F**) A673 cells in hypoxia; (**G**) SK-ES-1 cells in normoxia; (**H**) SK-ES-1 cells in hypoxia (*** = *p* < 0.001, *t*-test). NT—nontreated controls.

The DPP4 inhibitor linagliptin (LG) consistently and significantly decreased the total number of EwS cells in culture assessed by crystal violet (CV) staining. For A673 cells, LG decreased CV staining by 8.3% in normoxia (Figure 5A, *p* < 0.01, *t*-test, *n* = 15 per condition from three independent replicates) and by 9.3% in hypoxia (Figure 5B, *p* < 0.01, *t*-test, *n* = 10 per condition from two independent replicates). For SK-ES-1 cells, LG decreased CV staining by 9.1% in normoxia (Figure 5C, *p* < 0.05, *t*-test, *n* = 15 per condition from three independent replicates and 6.0% in hypoxia (Figure 5D, *p* < 0.05, *t*-test, *n* = 10 per condition from two independent replicates).

The proportion of dead cells (stained with ethidium homodimer-1) was unaltered by LG for A673 cells in normoxia or hypoxia (Figure 5E,F) and SK-ES-1 cells in normoxia (Figure 5G). However, in SK-ES-1 cultures treated with LG in hypoxia, cell death was 28.2% higher (Figure 5H, *p* < 0.001, *t*-test, *n* = 10 per condition from two independent replicates). These data demonstrate consistent effects of LG on reducing EwS cell number with cell and condition-specific effects on cell survival.

### 2.7. Role of DPP4 and NPY in THP-1 Monocytes

**Figure 6:**
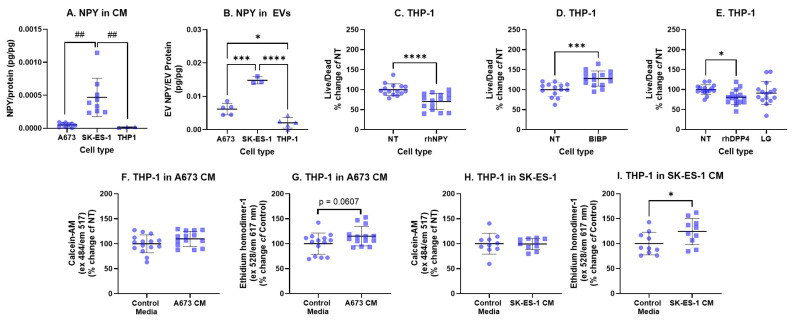
Effects of NPY-DPP4 signalling upon THP-1 monocytes. (**A**) NPY was measured in cell-conditioned media (CM) and normalised to total protein, revealing higher NPY secretion by SK-ES-1 cells compared with either A673 or THP-1 cells. (## = *p* < 0.01, Kruskal–Wallis with Dunn’s multiple comparison test). (**B**) NPY measured from extracellular vesicles (EVs) and normalised to total EV protein revealed higher NPY secretion into EVs from both A673 and SK-ES-1 cells compared to THP-1 cells (* = *p* < 0.05, *** = *p* < 0.001, **** = *p* < 0.0001, ANOVA with Dunnett’s multiple comparison test). (**C**) THP-1 viability was decreased with 1ng/mL rhNPY (**** = *p* < 0.0001, *t*-test). (**D**) Conversely, pharmacological blockade of NPY1R with BIBP 32226 increased THP-1 viability (*** = *p* < 0.001, *t*-test). (**E**) 20 U/mL rhDPP4 decreased THP-1 viability (* = *p* < 0.05, ANOVA with Dunnett’s multiple comparison tests), while blockade of endogenous DPP4 with 1 ng/mL linagliptin had no effect (*n* = 15 wells per condition from three independent experiments). (**F**,**G**) THP-1 cells incubated in A673 CM exhibited no significant differences in live/dead staining. THP-1 cells incubated in SK-ES-1 CM exhibited no change in (**H**) live cell staining, but an increase in (**I**) dead cell staining (* = *p* < 0.05, *t*-test). NT—nontreated controls.

In THP-1 cells, *NPY* transcript levels were unchanged following exposure to hypoxia but were decreased by LPS (Supplementary Figure S4A). NPY protein levels in THP-1 cell lysates, conditioned media (CM), and extracellular vesicles (EVs) remained uniformly low (Supplementary Figure S4B–F). Basal NPY secretion into CM was 1.2 ± 0.7 pg/mL, and when normalised to total protein, secretion from THP-1 cells was significantly lower than that from SK-ES-1 cells (Figure 6A). NPY secretion via EVs from THP-1 cells was also significantly lower compared with those from either A673 or SK-ES-1 cells (Figure 6B).

Although *DPP4* transcript levels were decreased in THP-1 cells with LPS treatment (Supplementary Figure S4A), DPP4/total protein levels in THP-1 cell lysates at 7.2 ± 3.2 ng/ng were not altered by hypoxia or LPS treatment (Supplementary Figure S5B), while DPP4 enzymatic activity was below the limit of detection. Despite this, DPP4 activity was measurable in THP-1 CM at 2.1 ± 1.3 µU/µg, which was not significantly altered under hypoxia (Supplementary Figure S5C) and was not significantly different to basal secreted DPP4 activity from A673 or SK-ES-1 cells. DPP4/protein levels in THP-1-derived EVs were 1.1 × 10^−4^ ± 2.1 × 10^−4^ ng/ng of DPP4/EV protein and were not altered by hypoxia or LPS (Supplementary Figure S5D) and were not significantly different from those in EVs derived from A673 or SK-ES-1 cells. DPP4 enzymatic activity was not detectable in THP-1-derived EVs. These results indicate low-level secretion of active DPP4 from THP-1 cells.

THP-1 viability, assessed using the live/dead assay, was decreased by rhNPY (Figure 6C) and increased by the NPY1R antagonist BIBP 3226 (Figure 6D). This is consistent with *NPY1R* transcript expression, which was present in THP-1 cells under normoxia and did not change under hypoxia. Although exogenous rhDPP4 also reduced THP-1 viability, inhibition of endogenous DPP4 with linagliptin had no effect (Figure 6E), likely reflecting insufficient endogenous DPP4 activity to inhibit. When THP-1 cells were incubated with conditioned media (CM) collected from A673 cells, live/dead staining did not show any significant changes (Figure 6F,G). When incubated with SK-ES-1 CM, live cell staining was not changed (Figure 6H), while dead cell staining was increased (Figure 6I, *p* < 0.05, *t*-test).

Collectively, these findings indicate that Ewing sarcoma cells, rather than monocytes, are the predominant source of NPY and DPP4. In contrast to their effects on EwS cells, where viability is increased, these factors act to reduce THP-1 monocyte viability.

## 3. Discussion

The principal aim of this study was to investigate the NPY-DPP4 signalling axis in Ewing sarcoma (EwS) and its interaction with monocytes, with the ultimate goal of identifying potential therapeutic targets that could be used for treatment with repurposed drugs. To distinguish effects that are cell-specific from those broadly applicable, we used two EwS cell lines, A673 and SK-ES-1, which are transcriptionally equidistant to primary EwS tumours (Supplementary Table S1). Experiments were conducted in both normoxia and hypoxia, given the known modulatory effects of hypoxia on NPY signalling [31,32].

We observed that both EwS cell lines secrete NPY, either directly into conditioned medium (CM) or via extracellular vesicles (EVs), suggesting autocrine signalling. Secretion from A673 cells is a novel finding that aligns with previous reports of SK-ES-1 secretion [14]. This may contribute to maintaining EwS cell viability. The association of NPY with EVs is particularly noteworthy, as it may stabilise NPY against enzymatic degradation by DPP4, prolonging its bioavailability. This is consistent with clinical observations of a very short systemic NPY half-life [33]. Exogenous rhNPY consistently increased EwS cell viability in both normoxia and hypoxia, with specific effects on the cell cycle: increasing S-phase cells in A673 while decreasing G2-phase cells, suggesting accumulation of viable cells in the synthesis phase. This finding corresponds to a previous study on SK-N-BE neuroblastoma cells, where NPY also increased the proportion of cells in S-phase [34].

DPP4 was also secreted by both EwS cell lines into CM and EVs. Basal levels were similar across cell types, but hypoxia induced cell-specific alterations: in SK-ES-1, hypoxia decreased DPP4 activity, whereas in A673, transcript expression increased, but without corresponding enzymatic activity. This disconnect suggests multiple roles for DPP4: enzymatically active DPP4 in CM can cleave NPY, whereas EV-bound DPP4 is inactive, potentially regulating EV uptake and intercellular signalling rather than enzymatic processing. Exogenous rhDPP4 increased live cell staining while inhibition of endogenous DPP4 with linagliptin consistently decreased EwS cell number and viability, particularly under hypoxia in SK-ES-1 cells, highlighting DPP4 as a potential therapeutic target. It is worth noting that DPP4 has multiple substrates, so it is possible that some of the effects of rhDPP4 could be mediated by substrates other than NPY. However, linagliptin is a highly specific antagonist to DPP4, and the observed effects of rhDPP4 are consistent with NPY cleavage.

It is noteworthy that although linagliptin decreased crystal violet staining of EwS cells in both normoxia and hypoxia, there was only a concomitant increase in cell death for SK-ES-1 cells in hypoxia. The lack of any increase in cell death concomitant with decreased crystal violet labelling in the other conditions could be explained by reduced proliferation. Data in this study indicates that DPP4 is a driver of EwS cell viability, as indicated by effects of exogenous rhDPP4 upon increased live cell staining and NADH metabolism (Figure 4). Concordantly, this suggests that inhibition of endogenously secreted DPP4 with linagliptin would decrease EwS cell viability. The additional finding that LG uniquely increases cell death of SK-ES-1 cells in hypoxia suggests that SK-ES-1 cells rely on endogenous DPP4 to maintain viability in hypoxic conditions, possibly as it cleaves NPY to activate NPY5R, which has already been found to be critical for SK-ES-1 cells in hypoxia [15].

*NPY1R* was expressed in both EwS cell lines, but NPY1R labelling was only apparent in A673 cells and its functional role differed. Blockade with the NPY1R antagonist BMS-193885 reduced total A673 cell number but had no effect on SK-ES-1 cells, suggesting differential receptor utilisation. That SK-ES-1 cells were able to respond to rhNPY in the absence of evidence for NPY1R expression or function indicates that another pathway must be responsible. SK-ES-1 cells secrete active DPP4, which has previously been shown to cleave full-length NPY to NPY _3-36_, which binds to NPY5R, a pathway that has been thoroughly characterised by Lu et al. linking NPY5R to metastasis in this line [15]. These results indicate that therapeutic targeting of NPY signalling must account for receptor expression heterogeneity across EwS subtypes.

Our data indicate that EwS cells secrete factors, including NPY and DPP4, that actively decrease the viability of THP-1 monocytes. rhNPY, rhDPP4 and EwS cell-conditioned media reduced THP-1 viability, whereas blockade of NPY1R increased it. Although EwS cell-conditioned media may contain additional factors that reduce THP-1 cell viability, its effects are consistent with those of NPY and DPP4, suggesting that these factors at least partially contribute to the observed effects of the conditioned media. These findings suggest that EwS cells may create an immunologically cold microenvironment by suppressing monocyte survival, contributing to poor immune infiltration in EwS tumours. Thus, targeting the NPY-DPP4 axis could have dual therapeutic benefits: reducing EwS cell viability while enhancing monocyte survival, potentially improving immune-mediated tumour control.

These findings identify several potential avenues for drug repurposing in EwS. Linagliptin consistently reduced EwS cell number and viability, particularly under hypoxia, suggesting potential utility of DPP4 inhibition in treating EwS tumours. Pharmacological inhibition of NPY1R with repurposed obesity treatments protects monocytes. Coupled with DPP4 inhibition, this could offer a dual strategy to counter both tumour progression and interactions with cells of the monocyte-macrophage lineage.

The presence of NPY and DPP4 in EVs, their autocrine signalling functions, and the differential effects under hypoxia emphasise the need for nuanced therapeutic strategies that account for microenvironmental heterogeneity and receptor-specific signalling.

**Scheme 1:**
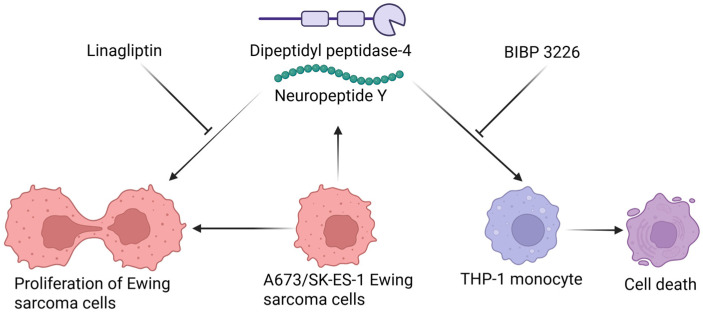
Ewing sarcoma (EwS) cells exhibit responses to NPY and DPP4 that are opposite to those observed in THP-1 monocytes. A673 and SK-ES-1 EwS cells both secrete DPP4 and NPY; treatment with DPP4 and NPY, contributing toward EwS viability, can be inhibited with linagliptin. Conversely, DPP4 and NPY decrease the viability of THP-1 monocytes, while pharmacological blockade of the NPY-sensitive NPY1 receptor with BIBP 3226 increases THP-1 viability. This study provides evidence for discrete targets in the NPY-DPP4-NPY1R signalling pathway that can independently modulate EwS cell and monocyte viability. Created in BioRender. Rumney, R. (2025) https://BioRender.com/g0e7bjt (accessed on 11 March 2026).

This study demonstrates that the NPY-DPP4 axis plays a multifaceted role in EwS biology, regulating cell viability, metabolism, and immune interactions in a cell- and hypoxia-dependent manner. Autocrine NPY and DPP4 signalling, coupled with EV-mediated persistence in biological fluids, may drive tumour progression and decrease monocyte viability. Therapeutic targeting of this pathway, through DPP4 inhibitors or NPY receptor antagonists, holds promise for both direct tumour suppression and enhancement of immune response in EwS as summarised in Scheme 1.

## 4. Materials and Methods

### 4.1. Celligner

The Celligner tool (https://depmap.org/portal/celligner/, accessed on 11 November 2025) [35] at the Cancer Dependency Map (DepMap) portal was used to identify the cell lines that most closely resemble the primary EwS tumour. Celligner used cell line RNAseq gene expression data from the DepMap Public 21Q1 file “DepMap DMC 21Q1 CCLE_expression_full.csv” and tumour gene expression data from the Treehouse Tumour Compendium V10 Public PolyA dataset available from the XENA browser.

### 4.2. Cell Culture Assays

Celligner [35] was used to calculate transcriptional distances between cell lines and specific tumour types to indicate the level of similarity (Supplementary Table S1). SK-ES-1 cells were selected for use as a bone-derived EwS cell line transcriptionally close to primary EwS tumours, which have previously been demonstrated to express the EWS-FLI1 transcript [14], and have featured in several recent high-profile studies into the roles of both NPY and DPP4 [13,15,31,36]. A673 cells were chosen as a comparator due to their highly similar transcriptional distance from the EwS tumour to that of SK-ES-1 cells. Although A673 cells were originally described as a rhabdomyosarcoma cell line, these have since been confirmed to be an extra-skeletal EwS that expresses the EWS-FLI1 fusion oncogene [37]. Because A673 cells had the most similar transcriptional distance from EwS tumours compared to SK-ES-1 cells, we selected A673 cells as the comparator cell type. Using more than one cell line in subsequent experiments allowed us to distinguish between results that are cell line specific and results that may apply to EwS more generally.

A673 and SK-ES-1 cells from ATCC were maintained as recommended by the supplier in 10% Foetal Bovine Serum (FBS) in DMEM and 15% FBS in McCoy’s 5A media, respectively (https://www.atcc.org/products/crl-1598 (accessed on 11 March 2026), https://www.atcc.org/products/htb-86 (accessed on 11 March 2026)) with 100 U/mL penicillin, 100 µg/mL streptomycin 37 °C at 5% CO_2_. For hypoxia, cells were kept at 1% O_2_. THP-1 cells were maintained as recommended by the supplier in RPMI-1640 with 0.05 mM β-mercaptoethanol and 10% FBS (THP-1—TIB-202|ATCC, Manassas, VA, USA). Each stock of heat-inactivated FBS (E.U.-approved, South America Origin, Gibco™, SKU 11550356, batch #2337973H) was sterile filtered through a 500 mL volume Filtropur V50, 0.1 µm pore size (Sarstedt, Nümbrecht, Germany) and stored in 50 mL aliquots at −80 °C before use.

For all experiments, EwS cells were seeded in media with FBS at a density of 60,000/cm^2^ allowed to attach for 24 h at 37 °C and 5% CO_2_. In a preliminary analysis, NPY protein and DPP4 activity were quantified in serial dilutions of heat-inactivated FBS, to establish the levels of each in cell-free media. We found that medium containing 2.5% FBS had 12.9 ng/mL NPY, 5% FBS had 27.8 ng/mL NPY, 10% FBS (equivalent to the ATCC recommended culture conditions for A673 cells) had 51.6 ng/mL NPY, while 15% FBS (equivalent to the ATCC recommended culture conditions for SK-ES-1 cells) contained 96.4 ng/mL NPY. Serum Neuropeptide Y (NPY) in healthy humans is typically ~0.6 ng/mL, while that of EwS patients is elevated to ~1 ng/mL [13], so even at the lowest concentration of FBS tested, the concentration of NPY protein was over 12 times higher than in EwS patient serum and 20 times higher than in healthy human serum.

DPP4 enzymatic activity was quantified from serial dilutions of heat-inactivated FBS in cell-free media. DPP4 activity in 2.5% FBS was 3.81 ± 0.85 µU/mL, in 5% FBS it was 8.1 ± 1.49 µU/mL, in 10% FBS it was 14.85 ± 0.89 µU/mL, and in 15% FBS it was 20.63 ± 1.67 µU/mL. DPP4 activity in human serum has been reported to be between 20 and 25 U/L [38,39].

The presence of supraphysiological levels of NPY and substantial DPP4 in FBS appears to be linked to decreased expression of relevant transcripts in the presence of FBS. FBS decreased *NPY* expression A673 cells in hypoxia (Supplementary Figure S6A); FBS decreased *DPP4* expression in A673 cells in hypoxia and SK-ES-1 cells in normoxia (Supplementary Figure S6C,D); FBS decreased *NPY1R* expression in A673 cells in normoxia and hypoxia (Supplementary Figure S6E); FBS also decreased *NPY5R* expression in A673 cells in hypoxia (Supplementary Figure S6G) and SK-ES-1 cells in normoxia (Supplementary Figure S6H). These differences also show that 48 h in KSR-containing media is sufficient to ameliorate effects of FBS on transcript expression.

Therefore, due to the potentially confounding presence of NPY and DPP4 in FBS, all experiments had to be carried out using media containing Knockout Serum Replacement (KSR) (Invitrogen, Paisley, UK) in place of FBS. This is consistent with previous studies where EwS stocks were maintained in 10–15% FBS, and experiments were carried out in low serum conditions [14,15]. Measurements of NPY and DPP4 levels in FBS stocks do not account for potential batch-to-batch variation. Therefore, we elected to replace FBS with KSR, thereby consistently eliminating FBS as a potential source of NPY and DPP4 that could confound experimental outcomes and improve reproducibility

A673, SK-ES-1 and THP-1 cells were seeded at 20,000 per well in 96-well plates directly with KSR-containing media. Treatments included 1 ng/mL Neuropeptide Y (NPY) (NM_000905) Human Recombinant Protein (CAT#: TP761100, Origene, Herford, Germany) corresponding to serum concentrations reported from patients with EwS between 0.940 ng/mL and 1.212 ng/mL [13]; 20 U/mL DPP4 (ab79138, AbCam, Cambridge, UK) corresponding to previous reports of 20 to 25 U/mL in human serum [38,39]; 1 ng/mL linagliptin (provided by Boehringer Ingelheim) corresponding to the recommended IC_50_ for the inhibition of DPP-4 activity [40], 5.9 nM BMS-193885 (B5063, Merck, Gillingham, UK) corresponding to the IC_50_ for NPY1R inhibition [29] and 7.2 nM BIBP 3226 trifluoroacetate [41]. All treatments were maintained for 48 h prior to analysis.

### 4.3. Cell Number Determination

For crystal violet (CV) assays, plates containing cells were fixed in 70% ice cold ethanol and air dried prior to staining with 0.5% *w*/*v* CV (Fisher Scientific, Loughborough, UK) in 20% methanol [42] solution for 20 min on an orbital shaker. Dye was removed, and plates were rinsed with dH_2_O until clear. Plates were air dried, cell-bound CV was solubilised in 100% methanol, and optical density was recorded at 590 nm.

### 4.4. Cell Viability Assay

The live/dead assay was carried out using the Invitrogen LIVE/DEAD™ Viability/Cytotoxicity Kit (Fisher Scientific, Loughborough, UK), for mammalian cells using the Fluorescence Microplate Protocol at Ex484/Em517 nm for Calcein and Ex528/Em617 nm for Ethidium homodimer-1 in the presence of DNA. Live/Dead data was normalised to baseline controls.

### 4.5. Migration Assay

Migration assays were carried out using transwell inserts for 24-well plates with an 8 µm pore size (#83.3932.800, Sarstedt, Leicester, UK) in accordance with protocols previously established specifically for EwS cells [43]. EwS cells were seeded into transwells at 60,000/cm^2^ in 24-well plates directly above treatment media with 10 µg/mL mitomycin C (BP2531, Fisher Scientific, Loughborough, UK) to prevent proliferation from confounding the results of the migration assay over a 24 h period. Transwells were wiped on the inside with cotton wool, fixed with 10% neutral buffered formalin for 20 min and quantified following CV staining. After images were captured, CV staining in transwells was solubilised in 100% methanol and optical density was recorded at 590 nm.

All plate reader assays were carried out using a SpectraMax i3x multi-mode microplate reader (Molecular Devices, Wokingham, UK).

### 4.6. Cell Cycle Analysis

For cell cycle analysis, paired cultures of cells grown in serum-free media under normoxia and hypoxia were harvested and fixed simultaneously, then stained with FxCycle PI/RNase solution (Fisher Scientific) following manufacturer’s instructions. Flow Cytometry was carried out on a Beckman Coulter Flow Cytometer with data analysis in CytExpert (Version 2.5.077) and Floreada.io (https://floreada.io/, accessed on 13 August 2024). The number of analysed events was 20,000 per sample and the gating strategy was set to default. Automated, gain independent fluorescence compensation settings were obtained using CytoFlex QC beads (Beckman Coulter, Amersham, UK; #C65719) before each run. Cell cycle analysis was carried out in Floreada.io based on the Watson Pragmatic model [44].

### 4.7. RNA Extraction, cDNA Synthesis and qPCR Analysis

Total RNA was extracted from cells using the RNEasy Plus Universal Mini kit (Qiagen, Manchester, UK, 73404) according to the kit manufacturer’s instructions. RNA quality and concentration were measured using a NanoDrop 1000 Spectrophotometer (Thermo Fisher Scientific, Altrincham, UK). RNA integrity was assessed using electrophoresis of 100 ng of total RNA in a 1% agarose gel (Sigma-Aldrich, Gillingham, UK; #A4718) in TAE buffer and visualisation of ribosomal RNA bands. Total RNA samples were converted to cDNA using SuperScript VILO cDNA Synthesis Kit (Invitrogen, Paisley, UK; #11754050) as per manufacturer instructions.

For qPCR, amplifications were performed in technical duplicates with 3–4 biological replicates, using 25 ng of cDNA per reaction with Applied Biosystems™ PowerTrack™ SYBR Green Master Mix (Fisher Scientific, Loughborough, UK) forward and reverse primers (Table 1) (Eurofins) and DEPC treated water (Fisher Scientific, BP561) as per manufacturer instructions, on 96-well plates using the QuantStudio™ 5 Real-Time PCR System (Thermo Fisher Scientific, Altrincham, UK). Data was normalised to *ACTB* with the ΔΔCT method.

qPCRs were carried out on cDNA from independent biological replicates (distinct passages for each cell line collected from independently ran experiments) ran in duplicate. The data points shown are the mean values of those technical duplicates for each biological replicate.

CT values for *ACTB* were consistent in both A673 and SK-ES-1 cell lines with no significant differences between normoxia and hypoxia (Supplementary Figure S7). *B2M* was used as the reference for THP-1 cells as it had greater stability in this cell line.

### 4.8. Immunofluorescent Labelling

Moreover, 5 mm glass coverslips (CS) with cells were fixed for 20 min at room temperature (RT) in 4% paraformaldehyde in PBS (pH 7.4). CS were rinsed with PBS prior to incubation with 0.1% Triton X-100 in PBS, then incubated for one hour in blocking buffer at room temperature (1% Bovine serum albumin (BSA), 10% normal goat serum (NGS), 0.1% Tween in PBS). CS were incubated with primary antibody against neuropeptide Y receptor type 1 (ab91262, AbCam, Cambridge, UK) at 5 µg/mL in blocking buffer overnight at 4 °C. After rinsing, CS were incubated with 10 ng/mL secondary antibody (Goat anti-rabbit Alexa 488) in blocking buffer for one hour at RT. CS were rinsed with PBS-tween and incubated with phalloidin red (1:40) and Hoechst (1:1000) at RT for 5 min before rinsing with PBS and mounting with Invitrogen prolong diamond antifade mounting agent.

The specificity of the primary anti-NPY1R antibody used (ab91262, AbCam) has been previously verified in a study investigating NPY1R expression in oral squamous cell carcinoma, where lentiviral knockdown and overexpression of NPY1R was confirmed using western blot [45]. Negative controls for secondary antibody with IgG control only did not show any punctate labelling similar to the NPY1R labelling (Supplementary Figure S3), and there was no non-specific labelling apart from some bleed-through of the nuclear Hoechst staining (Supplementary Figure S8).

### 4.9. NPY and DPP4 Protein Assays

Cell-conditioned media (CM) was collected from flasks, centrifuged at 300 g for 5 min to pellet down any non-adherent cells and sterile filtered through a 0.2 µm syringe filter. Extracellular vesicles (EVs) were isolated from CM following filtration through a 0.8 µm syringe filter, then using the exoEasy Maxi Kit (#76064, Qiagen, Manchester, UK) according to the manufacturer’s instructions. CM, EVs and cell lysates were snap frozen in liquid nitrogen immediately after collection for subsequent measurement of NPY and DPP4 protein and DPP4 enzymatic activity. NPY protein was quantified using the Human Neuropeptide Y (NPY) ELISA (#EZHNPY-25K, Merck, Gillingham, UK). DPP4 protein was quantified using the Human DPPIV/CD26 ELISA Kit (#RAB0147-1KT, Merck, Gillingham, UK). DPP-4 activity was measured with the Fluorometric Dipeptidyl peptidase IV Activity Assay Kit (ab204722, AbCam, Cambridge, UK). All assays were carried out according to the manufacturer’s protocols using a SpectraMax i3x multi-mode microplate reader (Molecular Devices, Wokingham, UK). All data was normalised to protein measured with the Pierce™ BCA Protein Assay Kits (Fisher Scientific, Loughborough, UK).

## 5. Conclusions

In conclusion, this study demonstrates that the NPY–DPP4 signalling axis plays a key role in regulating Ewing sarcoma (EwS) cell viability and suggests a mechanism for interaction with monocytes. Both A673 and SK-ES-1 EwS cells secrete NPY and DPP4, and stimulation of this pathway with recombinant proteins promotes tumour cell viability, with hypoxia further modulating these effects in a cell line-specific manner. In contrast, NPY and DPP4 decrease the viability of THP-1 monocytes, suggesting that EwS cells may exploit this signalling axis to promote tumour survival while suppressing immune cell viability within the tumour microenvironment. Pharmacological inhibition of DPP4 with linagliptin consistently reduced EwS cell number, highlighting the potential of targeting this pathway using repurposed therapeutics. Furthermore, inhibition of NPY1R with repurposed therapeutics could be used to improve immune cell viability for future immunotherapeutic interventions. Together, these findings identify the NPY–DPP4 axis as a potential dual therapeutic target capable of suppressing EwS growth while improving immune-supportive responses.

## Figures and Tables

**Table 1:** Primer Sequences.

Target	5′ to 3′ Sequence
*ACTB* forward	AGC CTT CCT TCC TGG GCA T
*ACTB* reverse	CTT CAT TGT GCT GGG TGC C
*B2M* forward	TGT CTT TCA GCA AGG ACT GGT
*B2M* reverse	GCG GCA TCT TCA AAC CTC CA
*DPP4* forward	TCT GTG GTG GTC TCC AAA CG
*DPP4* reverse	GGA TTC ACA GCT CCT GCC TTT
*NPY* forward	GTT CCC AGA ACT CGG CTT GA
*NPY* reverse	GCA GAA TTC AGC ACA GTG GC
*NPY1R* forward	CCA CTC TCC TCT TGG TGC TG
*NPY1R* reverse	GAG CCA GCA GAC TGC AAA TG
*NPY5R* forward	CTG GAT CAG TGG ATG TTT GGC
*NPY5R* reverse	GGA AGG GGA GAA CAG ATG GC

## Data Availability

The original contributions presented in this study are included in the article/Supplementary Material. Further inquiries can be directed to the corresponding author.

## Supplementary Materials

The following supporting information can be downloaded at https://www.mdpi.com/article/10.3390/ijms27062731/s1.

## Abbreviations

The following abbreviations are used in this manuscript:

DPP4	Dipeptidyl peptidase-4
EwS	Ewing sarcoma
LG	Linagliptin
NPY	Neuropeptide Y
NPY1R	Neuropeptide Y receptor 1
NPY5R	Neuropeptide Y receptor 5
