# Supplementary material for: Targeting Neuropeptide Y/DPP4 Signalling Suppresses Ewing Sarcoma Survival and Improves Monocyte Viability
Source: Int J Mol Sci. 2026 Mar 17;27(6):2731. doi: 10.3390/ijms27062731 (PMC13026981; doi:10.3390/ijms27062731)
Supplement: Supplementary file 1 [file ijms-27-02731-s001.zip › ijms-4127189-supplementary.pdf]

International Journal of
Molecular Sciences

MDPI

Supplementary figures

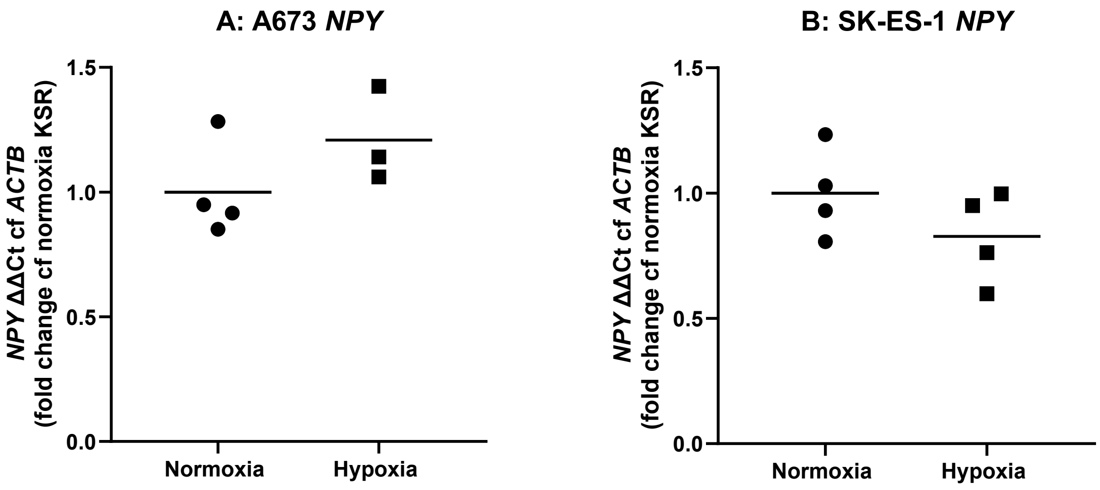

Supplementary Figure S1. Expression of *NPY* in EwS cells. *NPY* transcript expression was not significantly altered by hypoxia in (A) A673 and (B) SK-ES-1 cells.

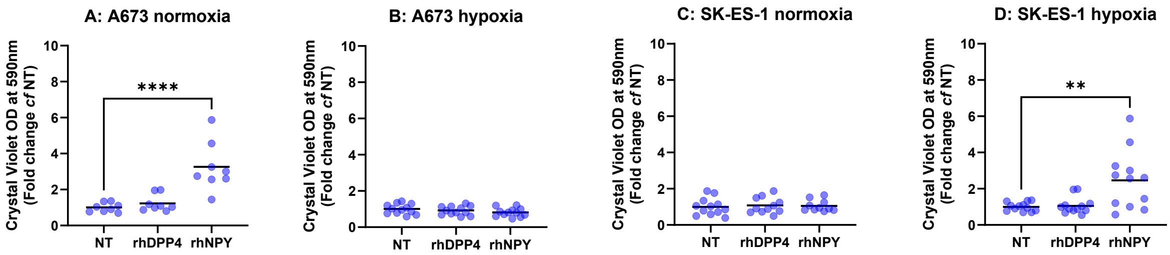

Supplementary Figure S2: Non-significant effects on cell migration

rhDPP4 had no effect on A673 or SK-ES-1 cell migration in either normoxia or hypoxia measured using the transwell assay (A-D). Significant effects of rhNPY upon (A) A673 migration in normoxia and (D) SK-ES-1 migration in normoxia are in the main manuscript (Fig. 2O and 2P respectively). rhNPY had no effect on (B) A673 migration in hypoxia or (C) SK-ES-1 cells in normoxia.

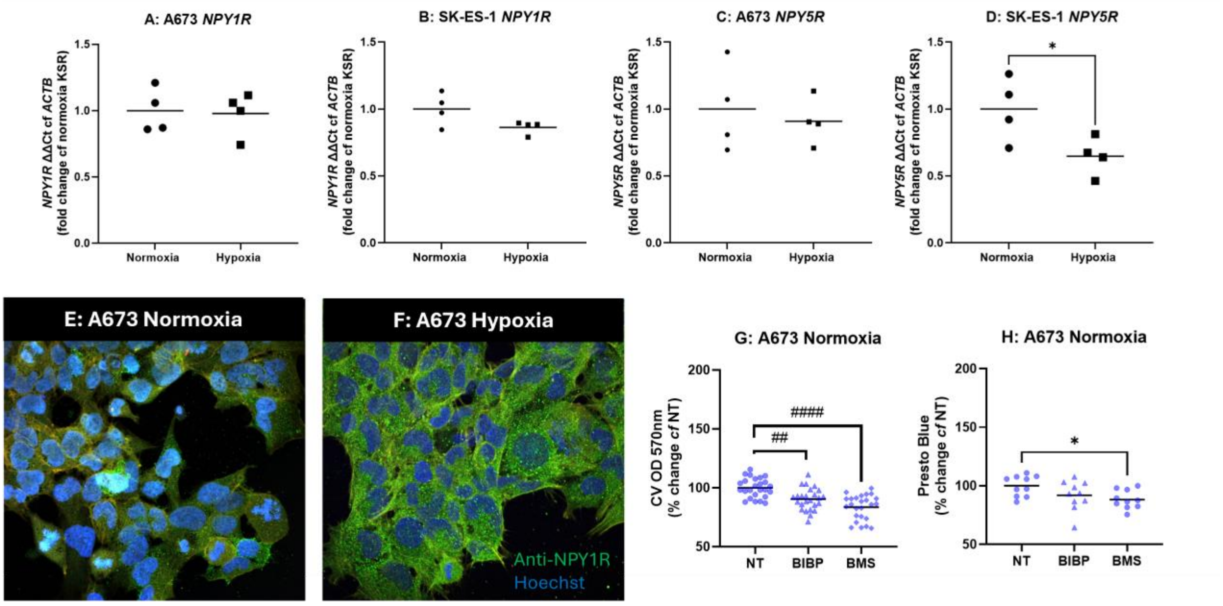

Supplementary Figure S3. Expression of *NPY1R*, *NPY5R* and effect of NPY1R inhibition in EwS cells. *NPY1R* transcript expression remained unchanged by hypoxia in (A) A673 and (B) SK-ES-1 cells. *NPY5R* transcript expression remained unchanged by hypoxia in (C) A673 cells but was decreased in (D) SK-ES-1 cells (t-test, * = p < 0.05). Immunofluorescent labelling of NPY1R protein in A673 cells in (E) normoxia and (F) hypoxia. Effect of treatment of A673 cells with the NPY1R antagonists BIBP 3226 and BMS-193885 for 48 hours in serum free media upon (G) CV staining of A673 cells in normoxia (## = p <0.01, #### = p <0.0001, Kruskal-Wallis with Dunn's multiple comparison tests) and (H) effect on metabolism in normoxia (* = p < 0.01, ANOVA with Dunnett's multiple comparison tests).

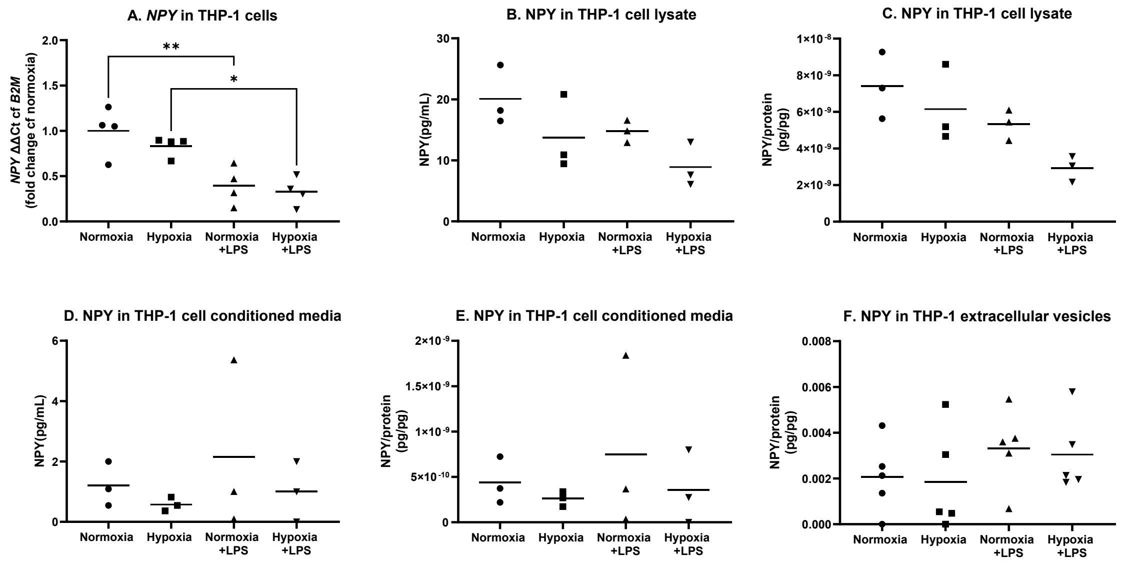

Supplementary Figure S4. Effects of hypoxia and LPS upon expression of *NPY* transcript and NPY protein in THP-1 cells. (A) *NPY* transcript expression was decreased in the presence of LPS in THP-1 cells (* = $p < 0.05$, ** = $p < 0.01$, ordinary one-way ANOVA with Holm-Šídák's multiple comparisons tests). (B) NPY protein levels from THP-1 cell lysates were not significantly changed, (C) even when normalised to protein. Similarly, NPY levels in cell conditioned media (D) were unaltered even when normalised to protein (E). NPY levels in EVs normalised to protein (F) also remained unaltered.

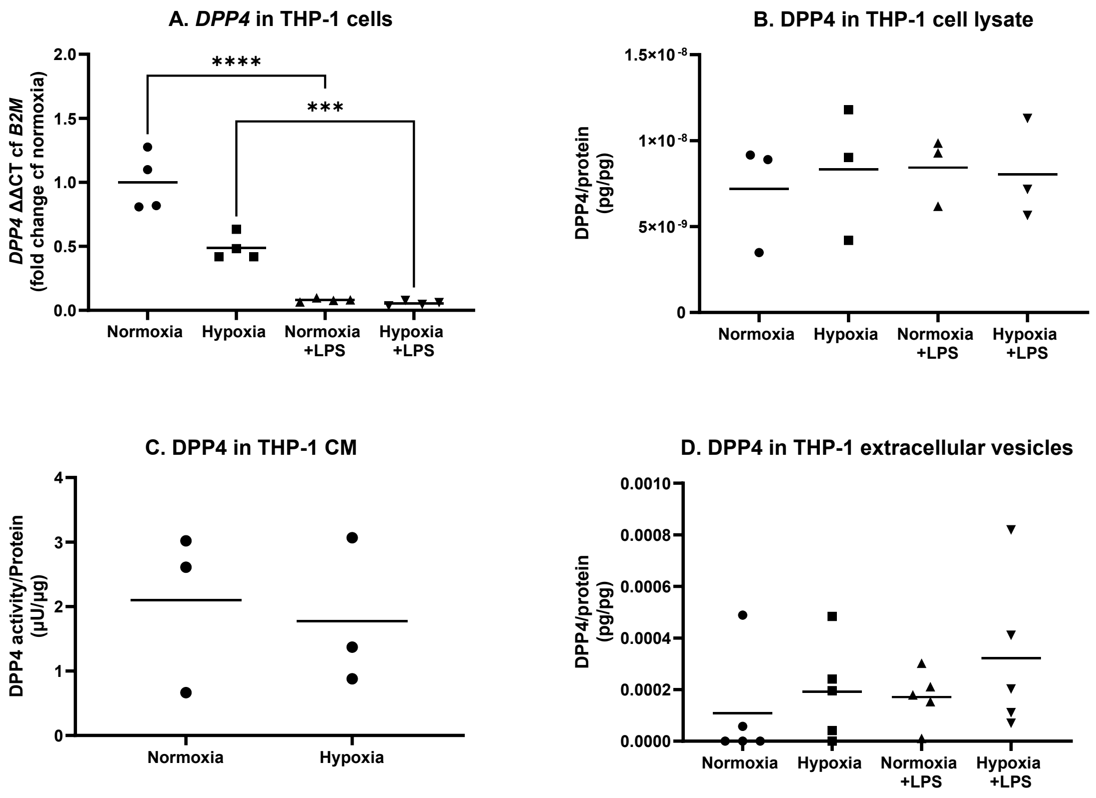

Supplementary Figure S5. Effects of hypoxia and LPS upon expression of *DPP4* transcript, DPP4 protein and DPP4 activity in THP-1 cells. (A) *DPP4* transcript expression was decreased in the presence of LPS in THP-1 cells (*** = $p < 0.001$, **** = $p < 0.0001$, ordinary one-way ANOVA with Holm-Šídák's multiple comparisons tests). (B) DPP4 protein normalised to protein were not significantly altered. (C) DPP4 activity from THP-1 cell conditioned media (CM) was not altered between normoxia and hypoxia. (D) DPP4 levels in EVs normalised to protein also remained unaltered.

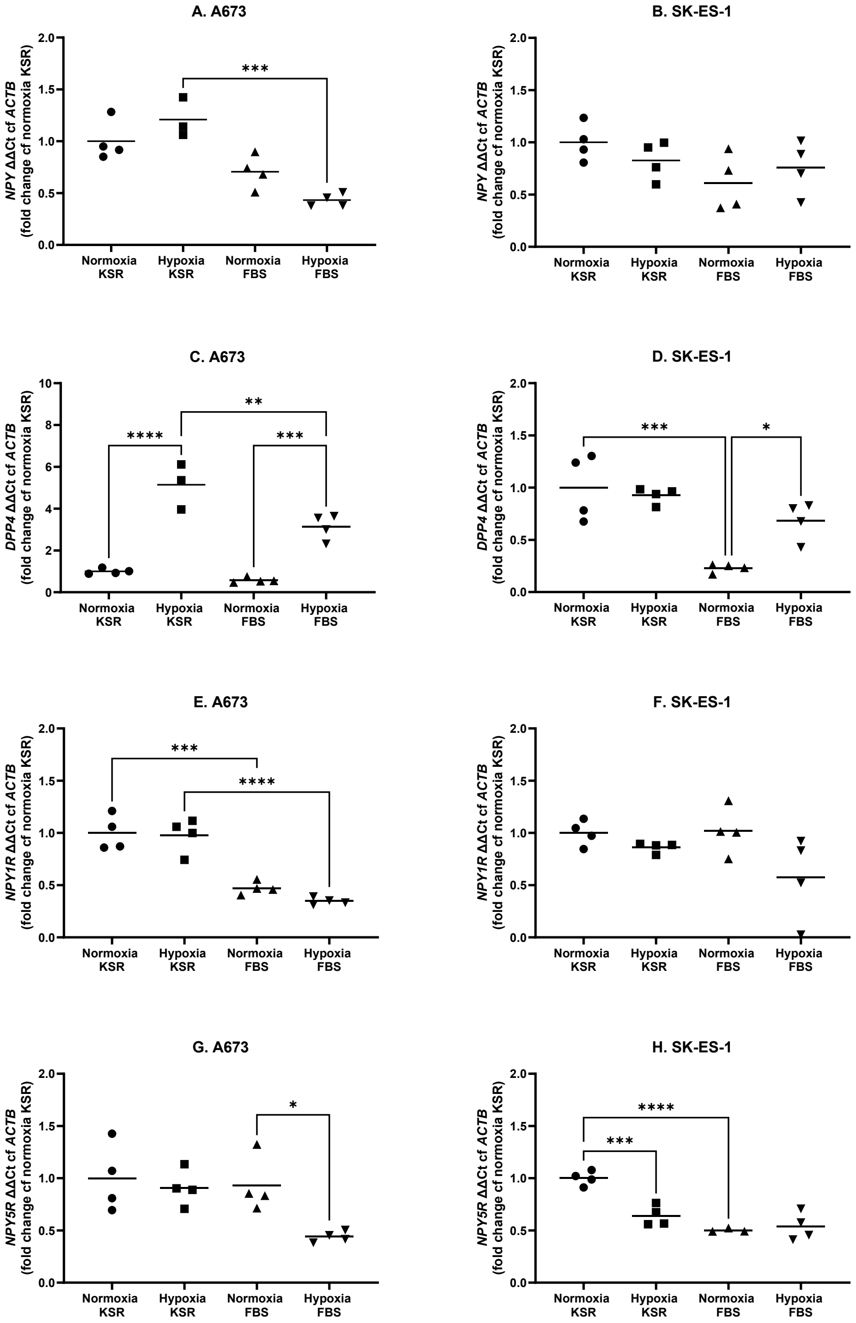

Supplementary Figure S6. Effects of FBS on transcript expression. Transcript expression for *NPY, DPP4, NPY1R* and *NPY5R* were recorded from A673 and SK-ES-1 cells in media containing either KSR or FBS in both normoxia and hypoxia. Each data point shown is the mean value of 2 technical replicates (wells across qPCR plates) from independent biological sample (cells from different passages and independent experiments). * = p <0.05, ** = p <0.01, *** = p <0.001and **** = p <0.0001, Ordinary One Way ANOVA with Šídák's multiple comparison post-tests.

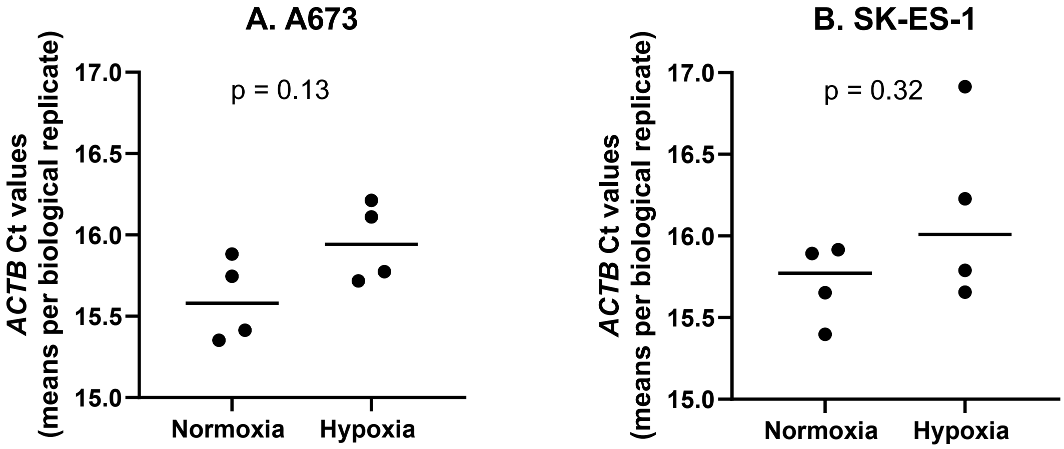

Supplementary Figure S7. ***ACTB*** **CT values.** *ACTB* transcript expression was recorded from four biological samples (cells from different passages and independent experiments) with 6 technical replicates of each (wells across qPCR plates). Data shown are the mean values for each biological replicate, demonstrating no significant differences between normoxia and hypoxia for A673 and SK-ES-1 cells.

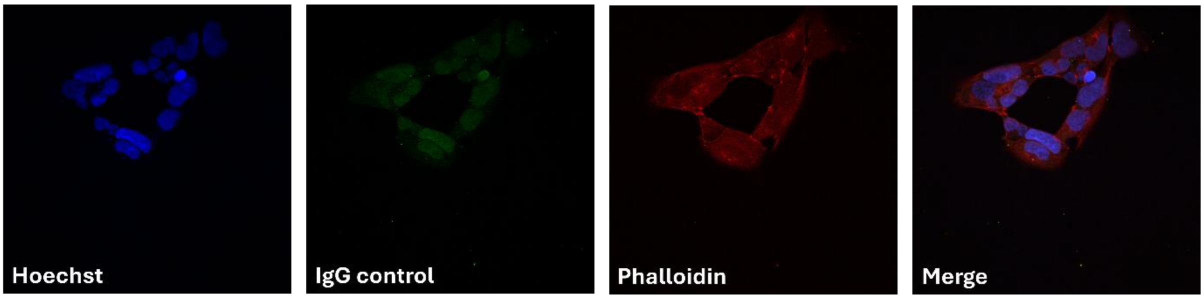

Supplementary Figure S8. Control immunofluorescent showing Hoechst (blue channel), IgG control with Goat anti-rabbit Alexa 488 secondary (green channel) and Phalloidin (red channel). Although there was some bleed through of the nuclear Hoechst on the green channel, there was none of the punctate labelling apparent with anti-NPY1R shown in Supplementary Figure S3.

Model Condition ID	Model Name	Sample Type	Distance	Lineage	Subtype
MC-000279-zY8v	EWS502	DepMap cell line	39.204	Bone	Ewing Sarcoma
MC-001030-DXsJ	CHLA218	DepMap cell line	39.394	Bone	Ewing Sarcoma
MC-000039-iKfU	SKNMC	DepMap cell line	39.994	Bone	Ewing Sarcoma
MC-000424-VRxU	TC71	DepMap cell line	40.413	Bone	Ewing Sarcoma
MC-001035-Wfke	CHLA99	DepMap cell line	42.58	Bone	Ewing Sarcoma
MC-001283-7e8M	TC106	DepMap cell line	42.646	Bone	Ewing Sarcoma
MC-001430-Lr6c	TC138	DepMap cell line	43.491	Bone	Ewing Sarcoma
**MC-000087-VZnM**	**SKES1**	**DepMap cell line**	**45.715**	**Bone**	**Ewing Sarcoma**
**MC-000052-uUW6**	**A673**	**DepMap cell line**	**46.053**	**Bone**	**Ewing Sarcoma**
MC-000499-mIS1	EW8	DepMap cell line	48.153	Bone	Ewing Sarcoma
MC-001034-dKi9	CHLA9	DepMap cell line	50.54	Bone	Ewing Sarcoma
MC-001192-OhhV	SKNEP1	DepMap cell line	51.153	Bone	Ewing Sarcoma
MC-001431-lLsj	TC205	DepMap cell line	53.077	Bone	Ewing Sarcoma
MC-001427-D9V7	CCLFPEDS0007T	DepMap cell line	54.136	Bone	Ewing Sarcoma
MC-001029-lvBN	CHLA10	DepMap cell line	55.011	Bone	Ewing Sarcoma
MC-001428-h8A5	CCLFPEDS0010T	DepMap cell line	55.498	Bone	Ewing Sarcoma
MC-001032-G8OH	CHLA32	DepMap cell line	56.881	Bone	Ewing Sarcoma
MC-001038-0vXG	COGE352	DepMap cell line	57.184	Bone	Ewing Sarcoma
MC-000041-uPBf	RDES	DepMap cell line	60.354	Bone	Ewing Sarcoma
MC-001022-4eAr	CBAGPN	DepMap cell line	60.836	Bone	Ewing Sarcoma
MC-001193-ObM2	SKPNDW	DepMap cell line	63.278	Bone	Ewing Sarcoma
MC-002780-ddZi	NCCES1C1	DepMap cell line	65.751	Bone	Ewing Sarcoma
MC-000391-CEkf	MHHES1	DepMap cell line	65.878	Bone	Ewing Sarcoma
MC-000210-6UEs	CADOES1	DepMap cell line	87.151	Bone	Ewing Sarcoma

Supplementary Table S1. Celligner distance values from cell lines to primary EwS tumour showing the near equidistance of SK-ES-1 and A673 cell lines from primary EwS tumour (https://depmap.org/portal, accessed on 11-November-2025).
